# The Role of Eye Movements in the Process of Silicone Oil Emulsification After Vitreoretinal Surgery

**DOI:** 10.3390/bioengineering11111081

**Published:** 2024-10-29

**Authors:** Irene Nepita, Camilla Brusati, Libero Liggieri, Francesca Ravera, Mariantonia Ferrara, Alessandro Stocchino, Mario R. Romano, Eva Santini, Rodolfo Repetto

**Affiliations:** 1 Consiglio Nazionale delle Ricerche-Institute of Condensed Matter Chemistry and Technologies for Energy (CNR-ICMATE), Via de Marini 6, 16149 Genoa, Italy; libero.liggieri@cnr.it (L.L.); francesca.ravera@cnr.it (F.R.); eva.santini@cnr.it (E.S.); 2 Department of Civil, Chemical and Environmental Engineering, University of Genoa, Via Montallegro 1, 16145 Genoa, Italy; brusaticamilla@gmail.com (C.B.); rodolfo.repetto@unige.it (R.R.); 3 Department of Medical and Surgical Specialties, Radiological Sciences and Public Health, University of Brescia, Viale Europa 11, 25123 Brescia, Italy; mariantonia.ferrara@gmail.com; 4 Department of Civil and Environmental Engineering, The Hong Kong Polytechnic University, Hung Hom, Hong Kong, China; alessandro.stocchino@polyu.edu.hk; 5 Department of Biomedical Sciences, Humanitas University, Pieve Emanuele, 20072 Milan, Italy; mario.romano.md@gmail.com

**Keywords:** eye movements, silicone oil, emulsion, vitreoretinal surgery, albumin

## Abstract

Emulsification is a feared and common complication of the use of silicone oil (SO) as tamponade fluid after vitrectomy as it potentially associated with significant risks to ocular health, including elevated intraocular pressure (IOP), glaucoma, corneal and retinal changes. The aim of this study was to investigate the role and interplay of physical factors on the formation of SO emulsion. Experiments were performed in a model of the vitreous chamber with a realistic shape, filled with SO and an aqueous solution containing different concentrations of albumin, an endogenous protein known to modify the interfacial properties between SO and aqueous solutions. The model was subjected to harmonic and saccadic rotations and kept at body temperature. Results indicated that no emulsions were detected in the absence of albumin in the aqueous solution, while the presence of the protein facilitated emulsion formation, acting as a surfactant. Mechanical energy from eye movements was also found to be a key mechanism to produce emulsification, with higher mechanical energy provided to the system leading to smaller droplet sizes. The emulsions formed were stable over extended times. This study highlights the complex interplay of factors influencing SO emulsification in the vitreous chamber. A better understanding of the mechanisms underlying SO emulsification is crucial for developing strategies to mitigate SO emulsion and the related complications.

## 1. Introduction

Pars plana vitrectomy (PPV) is the surgical procedure of choice to treat several vitreoretinal pathologies. Silicone oil (SO) is commonly used as long-term intraocular tamponade for the management of complex diseases, such as complicated retinal detachment [1]. However, multiple SO-related ocular complications have been reported so far [1]. In particular, the formation of intraocular SO emulsion is highly undesirable and appears to have a crucial role in complications affecting nearly all ocular structures, such as acute and chronic changes in intraocular pressure (IOP), optic neuropathy, retinal and corneal alterations, cataract, and extraocular extension [1,2]. In addition, SO emulsification appears to be strictly related to intraocular inflammation [1]. Indeed, emulsified SO droplets can be phagocytised by macrophages, microglial cells and retinal pigment epithelium (RPE) cells, triggering an inflammatory response, that can, in turn, promote further SO emulsification due to the release of endogenous proteins in the intravitreal aqueous phase, able to modify rheological interfacial properties between aqueous solution and SO [3]. In this regard, it has been demonstrated that various blood constituents, such as lymphocytes, plasma, serum, red blood cells, haemoglobin, fibrinogen, fibrin, γ-globulins, plasma lipoproteins and purified HDL- apolipoproteins, are bio-molecules able to act as emulsifiers/surfactants for SO when dissolved in aqueous solution, adsorbing at the water-oil interface and modifying its mechanical interfacial properties [4,5,6]. Nepita et al. [7] demonstrated that the presence of two serum proteins, albumin and γ-globulins, in an aqueous solution had a significant effect on the interface SO-aqueous resulting in emulsions stable on the time scale of months. Albumin was specifically chosen because it is the most abundant protein in blood serum, making it a representative model for testing the emulsification process. Previous works indicated that the preliminary emulsification results obtained using albumin and γ-globulins were not significantly different [7], supporting the use of albumin as a representative model for testing the emulsification process.

Besides the presence of surface active species, the formation of stable emulsions is related to input of mechanical energy into the system [8]. In the presence of surfactant molecules, such as albumin, even a small amount of mechanical energy could be sufficient to break the interface into small droplets, which, in addition, are stable against coalescence [7]. Thus, a major factor influencing SO emulsification is represented by eye movement as shear stresses at the SO-aqueous interface generated during eye rotations (such as saccadic motions and REMs) can destabilise the interface [9,10,11,12]. Fluid dynamics in the vitreous chamber produced by eye rotations has been studied with numerical models [13,14,15,16,17,18,19], experimental approaches [20,21,22], and in-vivo measurements [23,24], demonstrating that the flow field induced by eye rotations has a complicated three dimensional structure.

Recent experimental studies investigated the motion of SO partially filling a model of the vitreous chamber [9,10], the shape of the interface SO-aqueous solution in the eye [25], the dynamics induced by eye rotations [26] and the mechanisms relating shear flow generated by eye rotations with the breakdown of the SO-aqueous interface [27]. These studies highlight the possible key role that eye rotations can have in the generation of emulsions in the eye. Wang et al. [11,12] performed experiments in a spherical model of the eye, partly filled with SO and partly with a water solution containing non endogenous surface-active molecules and subjected to a sequence of rectangular pulses. Interestingly, the formation of a bulk emulsion was never observed, but droplets were detected at the triple line of contact between the interface and the model solid wall [11,12].

Aim of this experimental work was to investigate the role and interplay of eye rotations and albumin concentration, which, as discussed above, are considered two major factors influencing SO emulsification.

## 2. Materials and Methods

### 2.1. Experimental Setup

**Figure 1:**
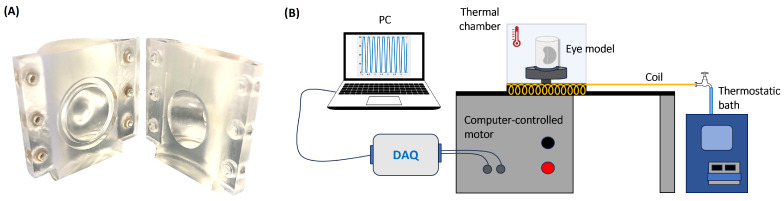
(**A**) Picture of the unassembled eye model; (**B**) sketch of the experimental apparatus.

The eye model has been specifically developed to simulate the real physiological conditions, in terms of temperature and geometry. The model was 3D printed, using Clear Resin 1l (RS-F2-GPCL-04), and it consists of a cylindrical container divided into two identical halves, with an internal cavity that reproduces the human vitreous chamber at real scale (Figure 1A). To ensure sealing of the model, a suitable groove to accommodate an O-ring is present on both halves of the model. The vitreous chamber has a realistic geometry and it exhibits the indentation due to the presence of the crystalline lens [28,29,30]. The total volume of the cavity is ≈5.2 mL, in accordance with the recent results of Azhdam et al. [31].

The eye model was connected to a stepper motor able to reproduce user-defined time law of the angular position. The motor was remotely controlled using a National Instruments^©^ data acquisition system (DAQ) and a Labview^©^ interface (National Instruments Corporation, Austin, TX, USA) to generate the command signal and recording the actual motor shaft position (Figure 1B).

To perform the experiments at physiological temperature, the model and the support connected to the motor shaft, were enclosed into a thermostatic chamber, characterised by an insulated base (26×26 cm) and a removable Plexiglas covering box. The temperature inside the chamber was maintained at 35 °C by pumping water from a thermostatic bath (RC 6 CP, LAUDA Scientific GmbH, Lauda-Königshofen, Germany) through a copper serpentine placed within the chamber (Figure 1B).

### 2.2. Working Fluids and Eye Model Filling Protocol

Before each experiment the eye model was carefully cleaned, with standard procedures adopted in surface science laboratories, in order to avoid contamination from surface active molecules and possible impurities. After assembling the eye model, aqueous solution and SO were pumped inside through a hole located on the side of the model, while air flowed out through a second hole, located at top of the model. During surgery it is never possible to completely fill the vitreous chamber with SO, thus in the present experiments 20% of aqueous solutions were introduced in the eye model. Such solutions were prepared in a Dulbecco phosphate buffered saline (DPBS, Sigma-Aldric D8662 (Merck KGaA, Darmstadt, Germany)), simulating the in-vivo environment, and adding different concentrations of bovine serum albumin (Sigma, A2153-50G) equal to 0% (bare buffer), 1% and 5% of the blood concentration (which is ≈50 g/L). Albumin was chosen as this protein has a surface-active behavior on the water-oil interface, comparable to that of the mixture of albumin/γ-globulins [7], indicating that albumin has a greater influence on the interfacial behavior of molecules present in blood serum.

The filling phase required special attention, to avoid the premature formation of oil droplets. After assembling the empty eye model, we first injected the protein-containing aqueous solution, which filled only a small fraction of the vitreous chamber. We also ensured that the inner walls of the model were homogeneously wetted with the aqueous solution to provide the correct wettability properties, a step we considered close to the condition after fluid-air exchange in clinical practice. Following this, we very slowly injected the commercially available SO, RS-OIL 1000 cSt (Alchimia srl, Padua, Italy) into the remaining empty cavity, taking care to minimise emulsification during this phase.

Specific experiments were carried out to evaluate the effect of SO injection (without setting the eye model into motion) on the possible formation of droplets (see Table 1).

### 2.3. Simulation of Eye Movements

Two time laws for simulating eye rotations were considered: harmonic rotations (HR) and a sequence of saccadic rotations (SR) in opposite directions.

A harmonic law is the simplest way to represent a sequence of saccadic eye movements in both directions, with prescribed amplitude and duration. This first set of experiments was run imposing a frequency of 5 Hz and varying the amplitude from 20° to 50°. Each experiment had a total duration of 1 h.

A single saccadic rotation is very well represented by the following equation, which was proposed by Repetto et al. [21],

(1) $$\theta \left(t\right)=c_{0}+c_{1}t+c_{2}t^{2}+c_{3}t^{3}+c_{4}t^{4}+c_{5}t^{5},$$

where θ is the rotation angle with respect to a given direction and *t* is the time. The 6 coefficients in the above expression can be determined by using metrics of the saccadic rotation, as measured by Becker [32]. In particular, he reported that saccade duration *D* linearly increases with the amplitude *A*, according to the relationship

(2) $$D=D_{0}+dA.$$

where *A* is measured in degs, *d* assumes the value of 0.0025 s/deg and $D_{0}$ is ≈0.025.

A periodic signal consisting of a sequence of saccadic rotations in opposite directions has been constructed on the basis of Equation (1). Specifically, the signal is periodic and a single period consists of a saccade in the clockwise direction, a period of rest of duration D/2, a saccade in the counter-clockwise direction and a final additional resting period, again of duration D/2. Thus, the period of the signal is equal to 3D (see Figure S1 in the Supplementary Material). Since, according to Equation (2), saccade amplitude and duration are related to each other, once the saccade amplitude *A* is chosen the angular frequency 2π/(3D) can be calculated. Again the total duration of the experiment was set to 1 h.

In order to assess the repeatability of the experiments, all tests have been conducted twice, obtaining very similar results, in terms of the observed emulsion. All performed experiments are summarised in Table 1.

### 2.4. Microscope Image Acquisition

At the end of each experiment, the eye model was removed from its support and, 24 h later, the formed emulsion was extracted and characterised with a microscope quantitative analysis. A DVM6-M optical microscope (Leica microsystems GmbH, Hamburg, Germany), with a CMOS sensor (3664 × 2748 pixel) and equipped with PlanAPO FOV 3.60 objective, which guarantees a maximum resolution of 2366 lp/mm, was used to determine droplet distribution and size.

Images of the spherical cavity’s content were first captured at 200× magnification to locate the emulsion in the vitreous chamber. Once identified, samples were taken and analysed on microscope slides.

Coalescence tests were carried out to determine the type of emulsion formed (water-in-oil W/O vs. oil-in-water O/W). A droplet containing the emulsion was placed on a sterile lab slide in contact with a droplet of buffer or SO (see Figure S2 in the Supplementary Material) and the behavior of the system was observed.

For each experiment we performed a droplet size distribution analysis. Specifically, a small amount of the emulsion (about 0.4 mL) was diluted with the continuous phase in order to obtain a good spacing between the droplets, and was then placed between two sterile microscope slides, kept at 0.2 mm distance by a spacer. The volume of the collected sample was equal to ≈7.5% of the total volume of the vitreous chamber model and diluted with an amount of aqueous solution variable from case to case. Although the total volume sampled could not allow for an accurate quantification of the total amount of emulsion, the adopted method is reliable and reproducible in terms of droplet size distribution, which is one of the findings we focused on.

Using the Leica Application Suite X proprietary software, (LAS X V 3.0.4, Leica microsystems GmbH, Hamburg, Germany) micro-photos of the emulsions were taken, with different magnifications (700×, 900×, 1200× and 1800×) with resulting spatial resolution in the acquired image ranging from 0.2 to 0.7 μm. These spatial resolutions are comparable with the physical limits of the optical microscope. Owing to these resolution constraints, we limited our analysis to drops with diameters >1μm. For each sample, about 15 images were acquired, which was sufficient to cover the entire sample.

The images were then analysed through the same software, to determine droplets size distribution. A specific tool of this software allows the operator to identify all drops within an image and it returns the droplets diameter. Since SO droplets reflected the microscope light, droplet boundaries could vary from 1 to 2 μm in thickness. For this analysis the external diameter of the drops was considered.

The size of the observed droplets was analysed in term of size frequency distribution, using a bin size of 5 μm, which is significantly larger than the experimental error (which is of the order of 1 μm).

For each test, we also computed the ratio *S* between the surface occupied by the droplets and the total area of the acquired images. This quantity was computed as

(3) $$S=\frac{\sum_{k=1}^{N}\sum_{i=1}^{M_{k}}s_{k}^{\left(i\right)}}{\sum_{k=1}^{N}A_{k}},$$

where *N* is the number of considered images, $M_{k}$ is the number of droplets in the *k*-th image, $A_{k}$ is the area of the *k*-th image and $s_{k}^{\left(i\right)}$ is the area of the *i*-th droplet in the *k*-th image. The dimensionless number *S* allowed us to compare images obtained with different magnifications in terms of area occupied by the droplets.

The performed statistical analysis was based on the hypothesis that the drops observed on the focal plane of interest were sufficiently representative of the emulsion; this aspect was confirmed by the repeatability of the experiments.

## 3. Results

No emulsions were detected in the absence of endogenous proteins in aqueous solution; whereas, emulsified SO droplets formed in all the tests with aqueous solution containing albumin. Remarkably, this happened for all rotations imposed to the eye model and all albumin concentrations (1% and 5% of blood serum concentration). The results clearly indicate that even at a low concentration of albumin, significant emulsification occurs, due to a noticeable reduction in interfacial tension. This aligns with our previous findings [7], where albumin at both 1% and 5% was shown to facilitate stable emulsions. In general, we predominantly observed the formation of SO droplets in the region of the geometric indentation, which reproduces the crystalline lens. This is the site where the flow field induced by eye rotations has a complicated three-dimensional structure [15,16].

In experiments without eye motion, a very small amount of SO droplets was detected in the case of the highest concentration of albumin (5%) (see Figure S3 of the Supplementary Material), confirming that the emulsion observed in the experiments described in the following can almost entirely be attributed to the motion of the eye model.

Coalescence tests confirmed that all the emulsions were of SO in aqueous solution type (O/W) since we invariably observed that, the emulsion coalesces with the aqueous buffer; this did not happen when SO was added (see Figure S2 in the Supplementary Material).

We kept the formed emulsions in static conditions at room temperature for a week and did not observe any appreciable change in drop number and size, which shows that the emulsions are stable over long time scales.

**Figure 2:**
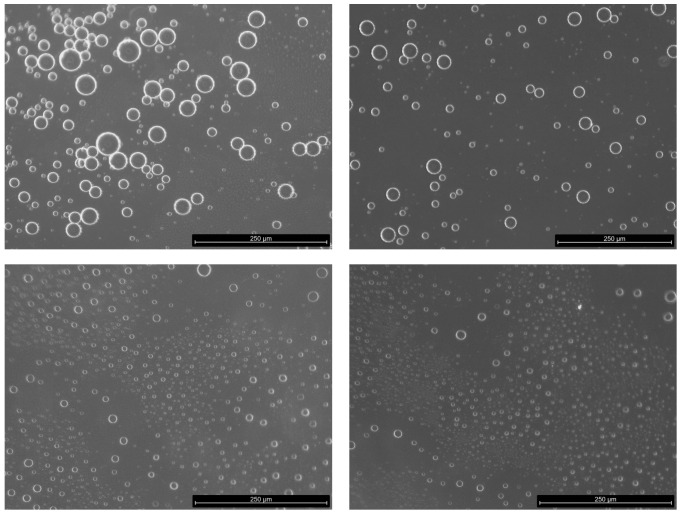
Examples of microscope images of the emulsion formed dilute with the continuous phase for droplet distribution analysis. HR experiment with albumin at 1% and *A* = 40°.

Figure 2 includes representative images acquired with the optical microscope, showing the presence of drops with different size, as well as sub-micrometric droplets, below the software resolution of about 1μm for the magnification used. Thus, droplet sizes <1μm are not included in the size distribution discussed below.

**Figure 3:**
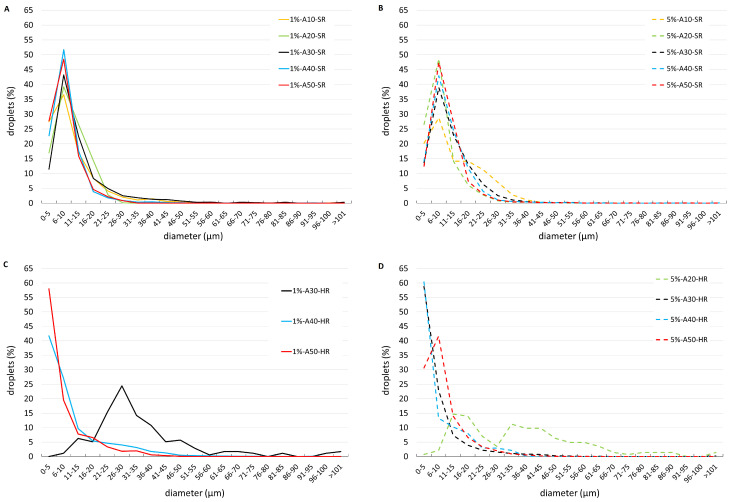
Comparison of droplets size distributions (in %) of emulsions obtained through SR experiments (**A**,**B**) and HR experiments (**C**,**D**), for all the investigated rotation amplitudes. Solutions containing 1% of albumin (**A**,**C**) and 5% of albumin (**B**,**D**). Different color bars correspond to different imposed amplitudes.

Figure 3 shows droplet distributions (in %) at the two investigated albumin concentrations and the two types of imposed rotation, SR (Figure 3A,B) and HR (Figure 3C,D). Droplet size distributions obtained with SR (Figure 3A,B) qualitatively resemble a Poisson distribution. The majority of drops has a very small diameter and the most frequent diameter class is 6 to 10 μm. No particular differences were observed between the two albumin concentrations tested (see also Figure S4 in the Supplementary Material). The distributions seem to suggest that increasing the amplitude of eye rotations leads to an increase in the number of small droplets, though differences between different rotation amplitudes are subtle and not all curves reported in the figures are ordered accordingly. The dependency on the eye rotations amplitudes is clearer for HR: SO droplets get smaller as the amplitude increases. This is because for HR the amplitude is changed keeping the frequency fixed, which is not the case for the SR, as explained in Section 2.3. For rotations amplitudes of $40^{\circ }$ and $50^{\circ }$ we did not manage to resolve the left, growing branch of the curves, which suggests that a large number of formed droplets were too small to be detected, likely due to the more intense motion imposed in the case of HR than that corresponding to SR (see also Figures S4 and S5 in the Supplementary material). In fact, by increasing the amplitude of HR while keeping the rotation frequency constant we obtained the generation of denser emulsions. We note that, in the case of large amplitude oscillations, the motion is too intense to represent physiological conditions. However, HR experiments have the value to clearly demonstrate that the intensity of eye movements has a key role in the process of droplet generation and that the higher the mechanical energy put into the system, the smaller the average droplet size, as shown by the black curve in Figure 3, which is displaced towards the left as the concentration varies from 1% to 5%, and almost collapses on the other curves, which correspond to rotations of larger amplitude.

**Figure 4:**
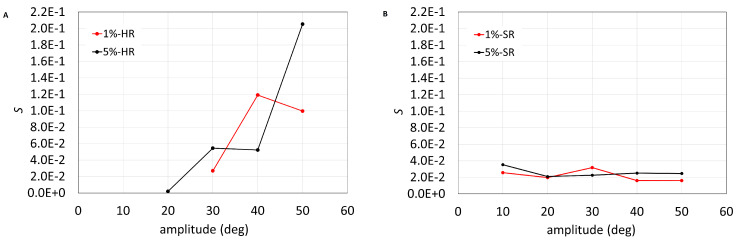

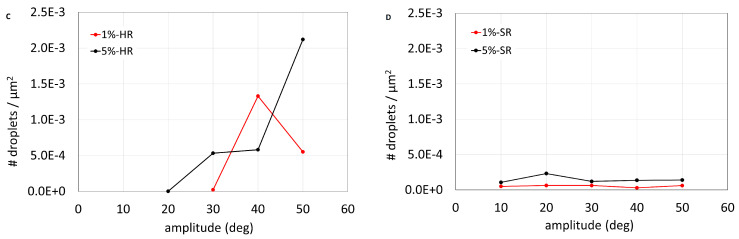
(**A**,**C**) HR experiments, (**B**,**D**) SR experiments. (**A**,**B**) Surface area occupied by droplets with respect to the area of the acquired images (*S*), as a function of the imposed rotation amplitude. (**C**,**D**) Total number of droplets per unit area as a function of the imposed rotation amplitude. Different colors correspond to different albumin concentration.

The results shown in the Figure 4, parameter *S* (A,B) and number of droplets (C,D) vs. amplitude of rotations, confirm the findings discussed above. In particular, the dimensionless parameter *S* and the droplet density grows with the rotation amplitude in the case of HR but not of SR. Figure 4 demonstrates that, with realistic SR movements, even at low concentrations of albumin (1%) the emulsification effect is strong.

## 4. Discussion

In this study we investigated the formation of SO emulsion in a vitreous chamber model, focusing on the influence of eye rotations, albumin concentration and their interplay. Although similar experiments have been previously conducted [11,12] our study presents some key differences. First, our model is not spherical but has a realistic geometry that closely matches that of the human vitreous chamber. Analytical [15,16] and experimental [22] works have shown that even a weak departure of the vitreous chamber shape from spherical has a very significant effect on vitreous motion, causing the formation of fluid circulations in the anterior part of the chamber, due to the change in curvature of the wall produced by the crystalline lens. Second, we kept the model at body temperature during all the experiments, which possibly has a major effect on the formation of an emulsion. In fact, the chemico-physical properties of SO-aqueous interface are strongly dependent on temperature [33]. Moreover, an increase in temperature is known to reduce the interfacial tension [34,35], thus facilitating emulsification. Third, we use aqueous solutions containing two different concentrations of albumin, due to the demonstrated major role on interfacial properties [7]. Finally, realistic saccadic rotations have been imposed on the eye model.

The eye model was set in motion following both simple harmonic time laws (HR) and also realistic sequence of saccadic rotations (SR). All experiments lasted an hour, thus consisting of a very long series of successive eye movements.

In the absence of albumin we never found formation of SO droplets in the aqueous solution, which confirms its key role in the emulsification process. We note that, though albumin is not normally present in the aqueous humor [36], its presence in the vitreous chamber after PPV is almost certainly unavoidable, owing to possible bleeding and the postoperative inflammatory processes [1].

In all experiments we followed the same SO injection procedure and we verified that the injection phase alone did not produce bulk emulsification but only a very small amount of droplets in the vitreous chamber. This confirms that the majority of observed drops in the experiments can be attributed to the mechanical energy generated by eye movements. We note that, in our experiments, we approximately replicate the clinical conditions of fluid-air exchange followed by SO injection. However, we took specific precautions to avoid emulsification during the filling phase and used filling needles different from those used in the real practice. We cannot exclude the possibility that, in surgical practice, some degree of emulsification may occur during the filling process itself due to the different dynamics involved. A more accurate replication of the surgical conditions, such as using a real vitrectomy machine, would be necessary to investigate this effect. Our approach was designed to focus on the emulsification induced by mechanical energy from eye movements, and to isolate this from any emulsification potentially caused by the filling phase.

We found with coalescence experiments that all the emulsions that formed during the test were O/W emulsions, in agreement with the Bancroft rule [37] (and not as hypothesised elsewhere) [38].

A different research group carried out similar experiments and did not observe the formation of bulk emulsions, but the generation of droplets only in the vicinity of the triple line of contact of the interface with the wall [11,12,39]. The authors provide sound physical explanation of how the movement of the triple line could cause the formation of SO droplets. The discrepancies with our results are likely to be attributed to the use of a model of the vitreous chamber with realistic geometry and kept close to body temperature. We acknowledge that using a single viscosity of SO (1000 cSt) could be seen as a limitation. SOs with higher viscosities, like 5000 cSt, demonstrated to be more resistant to emulsification in vitro [9,40]. However, we chose 1000 cSt as it is commonly used in the clinical practice and has already been shown to emulsify in the presence of endogenous proteins, as shown in previous experiments [7]. Finally, instead of non-endogenous, surface-active molecules [11,12], we employed an aqueous solution containing albumin, which has previously been shown to significantly affect the properties of the SO aqueous solution interface [7]. All the above ingredients make the formation of emulsions more likely.

The characterisation of the size distribution of the observed SO droplets showed that drops were of a very small size, with the class comprising the majority of drops typically being below 10 μm. Additionally, areas containing submicrometric droplets were observed. This is in line with previous studies demonstrating that small SO droplets (<2μm) represent the major component of SO emulsification in vivo [41,42]. Furthermore, the strong positive correlation between the number of small SO droplets (<2μm) and larger ones (7–30 μm), as reported by Chan et al. [41], reinforces the importance of our findings in enhancing the understanding of SO emulsion behavior and its implications for clinical outcomes. From a clinical point of view, emulsified SO microdropltes may be particularly dangerous as they can be phagocytosed by macrophages, resident microglial and RPE cells, thus, promoting the SO-related intraocular inflammation and, in turn, further SO emulsification [3]. In particular, an inflammatory granulomatous foreign-body reaction with epithelioid cells, induced by SO droplets phagocytosed by RPE cells has been reported [43]. In support of the interlink between SO and intraocular inflammation, the analysis of the aqueous levels of prostaglandin E2 and interleukin-1α in eyes treated with PPV and SO or heavy SO tamponade demonstrated that the inflammation grade significantly correlated with SO retention time [3]. It has also been suggested that proinflammatory cytokines accumulated in the fluid between the retina and the SO bubble may induce a local inflammation contributing to the development of proliferative vitreoretinopathy [44]. In addition, the detection of emulsified SO droplets within ocular structures of both anterior and posterior segment, support not only their ability to penetrate tissues, but also their relevant contribution to complications associated with SO use [41,45,46]. For instance, emulsification, inflammation and mechanical obstruction of the trabecular meshwork, appear to have a fundamental role in SO-related IOP elevation and secondary glaucoma [47]. The migration of emulsified SO droplets and the consequent contact with corneal endothelium may have a crucial role in SO-related corneal changes [48]. Epiretinal, intraretinal and subretinal emulsified SO droplets have been detected as hyperreflective dots on spectral domain-optical coherence tomography (SD-OCT) [49,50] and the SO-related thinning of inner retinal layers is one of the mechanisms potentially involved in SO-related vision loss [2]. Although it is well known that SO can lead to these potentially severe ocular complications, its role as long-term ocular endotamponade is still irreplaceable, in particular for complex rhegmatogenous retinal detachments [1]. Indeed, at the moment, there is no alternative compound offering the same tamponade profile as SO. In particular, perfluorocarbon liquids and semifluorinated alkanes are heavier than water [51]; in addition, the former is used as intraoperative tool due to its higher tendency to emulsify and the severe complications associated with its intraocular long-term use [1,52,53], whereas the used of perfluorohexyloctane (F6H8) as long-term intraocular tamponade has been limited by its significant tendency to emulsify [54]. On the other hand, fluorinated gases have a limited intraocular duration due to their spontaneous reabsorption and are not able to cause a compartmentalisation within the eye [55]. Finally, hyaluronic acid-based vitreous substitutes are still under development and investigation [56].

In the experiments in which the eye model was subjected to HR of large frequency and amplitude, SO drops became too small to be counted and measured with the optical techniques employed in this work. The general indication, is that the higher the amount of mechanical energy put into the system and the higher albumin concentration in the aqueous phase, the smaller the size of droplets formed. In line with this, SR sequences exhibited less variation in droplet size, as the increased saccade amplitude led to a corresponding decrease in frequency, reducing the shear stress and energy available for emulsification [21]. We note that we focused on HR and SR, as simple models of eye movements that generate shear stresses at the SO-aqueous interface. While additional movements, most notably REMs [57], could be simulated, we believe the idealised motions used already capture the essential dynamics leading to SO-emulsification. Moreover, in the present study, we used a minimal amount of aqueous solution to represent the fluid plausibly produced by the eye over time after surgery. While this volume was chosen to mimic post-operative conditions, it is plausible that an increased volume of aqueous solution, especially if containing endogenous proteins, could further enhance emulsification. In fact, a larger volume of fluid would likely increase the interface area between the SO and the aqueous phase, thus promoting more significant emulsification.

With regard to the influence of albumin concentration on emulsification, it was evident in both HR and SR cases. We observed that even at low concentrations of albumin (1%), a strong emulsification occurred. This behavior was consistent both with HR and SR experiments, confirming that albumin plays a critical role in promoting SO emulsification, even at minimal concentrations, as previously demonstrated in our earlier study [7]. The differences between 1% and 5% albumin concentrations were subtle, especially in the case of more realistic SR, suggesting that even very small amounts of proteins in the aqueous solution are sufficient to induce changes in the rheological properties of the interface, leading to emulsion formation.

Clinically, these results may highlight the relevance of surgery- and patient-specific findings in the development of SO emulsification. Indeed, the minimisation of intraoperative bleeding and an optimised control of inflammation after PPV might minimise the potential presence of albumin (as well as other blood serum proteins) into the vitreous chamber and, thus, contribute to lower the rate of postoperative SO emulsification. Finally, the verified stability over times of SO emulsion may support its potential to induce long-term detrimental effects.

## 5. Conclusions

Our study demonstrated that in the absence of proteins, no emulsification occurred, regardless of the eye movement conditions. However, when albumin was present in aqueous solution, even at low concentrations (1% of the blood concentration), emulsification always occurred. This finding underscores the role of proteins in modifying significantly the interfacial rheological properties and making the system SO-aqueous solution prone to emulsification. Moreover, we observed that the emulsions formed consist of very small droplets with diameters less than 10 μm. These small droplets may be particularly dangerous due to their ability to penetrate ocular tissues, potentially leading to intraocular inflammation and further emulsification. Finally, we confirmed that the emulsions formed were stable over times and of the oil-in-water type (O/W). These findings provide critical insights into the mechanisms of SO emulsification and its potential clinical risks. Future studies could explore the effects of other endogenous proteins, variations in SO viscosity and different model geometries, such as those of myopic eyes to develop more effective strategies to mitigate emulsification and its complications.

## Figures and Tables

**Table 1:** List of the performed experiments.

	Injection	Saccadic Rot. (SR)	Harmonic Rot. (HR)
**Albumin Conc. (%)**	**Amplitude (Deg)**	**Amplitude (Deg)**	**Amplitude (Deg)**
0	0	40	40
1	0	10, 20, 30, 40, 50	30, 40, 50
5	0	10, 20, 30, 40, 50	20, 30, 40, 50

## Data Availability

The raw data supporting the conclusions of this article will be made available by the authors on request.

## Supplementary Materials

The following supporting information can be downloaded at: https://www.mdpi.com/article/10.3390/bioengineering11111081/s1. Figure S1: Single period of the saccade signal used for the SR experiments with A imposed between 10° and 50°. Figure S2: Consecutive microscope acquired frames of the coalescence test. (A) DPBS droplet (right) positioned close to the droplet of emulsion (left); (B) Occurred coalescence between the two droplets. This allows to consider the emulsion as oil-in-water (O/W) type. To verify this result the same test has been performed positioning an oil droplet close to a droplet of emulsion. In this case, these two phases di not coalesce, confirming that the continuous phase of the emulsion is the DPBS solution. Figure S3: Comparison of droplets size distributions (in number of droplets) of emulsions obtained through SR experiments and with 5% of albumin concentration for all the investigated rotation amplitudes. Different color bars correspond to different imposed amplitudes. In the figure the droplets size distribution of drops observed after the injection phase is also reported. Figure S4: Comparison of droplets dimension distribution (in%) related to SR experiments with albumin concentration at 1% (continuous lines) and at 5% (dashed lines). Each panel reports the results obtained by imposing the same saccadic amplitude: (A) A = 10°, (B) A = 20°, (C) A = 30°, (D) A = 40°, (E) A = 50°. Results obtained with the two albumin concentrations resulted very similar for all the imposed rotation amplitudes. Figure S5: Example of microscope images of emulsions obtained through HR experiments with A = 50°. A significantly larger area occupied by SO droplets for each image can be observed with respect the images acquired with lower HR amplitudes for the same albumin concentration.
