# Supplementary material for: The Role of Eye Movements in the Process of Silicone Oil Emulsification After Vitreoretinal Surgery
Source: Bioengineering (Basel). 2024 Oct 29;11(11):1081. doi: 10.3390/bioengineering11111081 (PMC11591131; doi:10.3390/bioengineering11111081)
Supplement: Supplementary file 1 [file bioengineering-11-01081-s001.zip › bioengineering-3208964-supplementary.pdf]

The role of eye movements in the process of silicone oil emulsification after vitreoretinal surgery

Irene Nepita,[1] Camilla Brusati,[2] Libero Liggieri,[1] Francesca Ravera,[1]
Mariantonia Ferrara,[3] Alessandro Stocchino,[4] Mario R. Romano,[5]
Eva Santini,[1] and Rodolfo Repetto[2]

[1]CNR-ICMATE, Institute of Condensed Matter Chemistry and Technologies for Energy, Genova, Italy

[2]Department of Civil, Chemical and Environmental Engineering, University of Genoa, Genova, Italy

[3]Department of Medical and Surgical Specialties, Radiological Sciences and Public Health, University of Brescia, Brescia, Italy

[4]Department of Civil and Environmental Engineering, Hong Kong Polytechnic University, Hung Hom, Kowloon, Hong Kong, China

[5]Department of Biomedical Sciences, Humanitas University, Milan, Italy

August 29, 2024

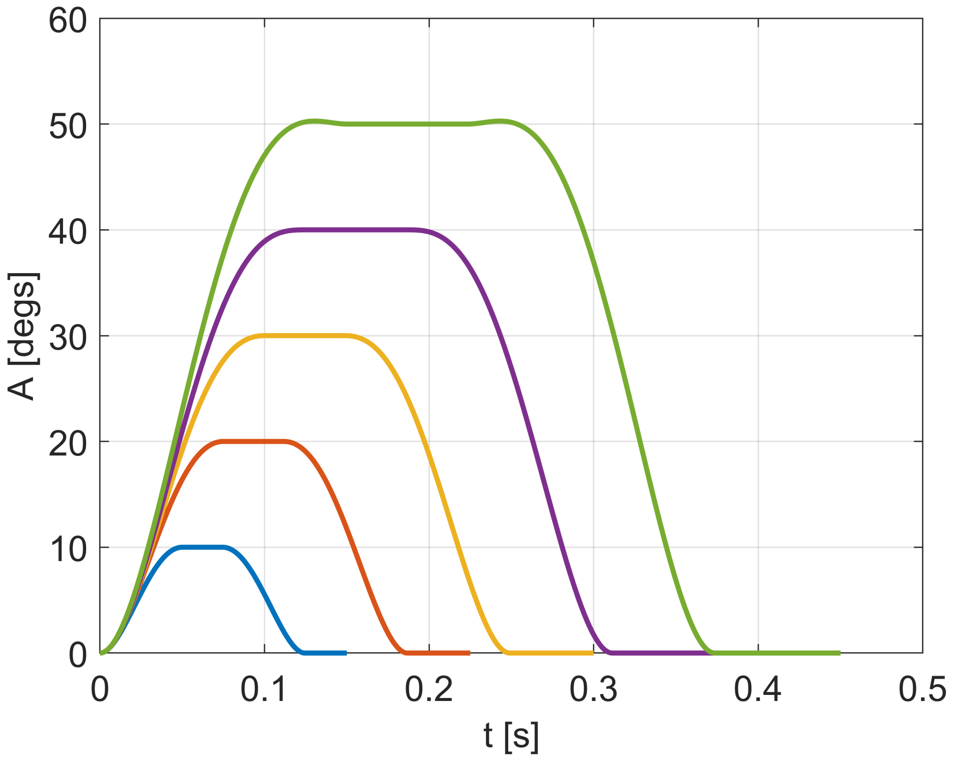

Figure S. 1: Single period of the saccade signal used for the SR experiments with A imposed between 10° and 50°. The period of the signal is equal to $3D$ and consists in a saccade in the clockwise direction, a period of rest of duration $D/2$, a saccade in the counter-clockwise direction and a final additional resting period of duration $D/2$.

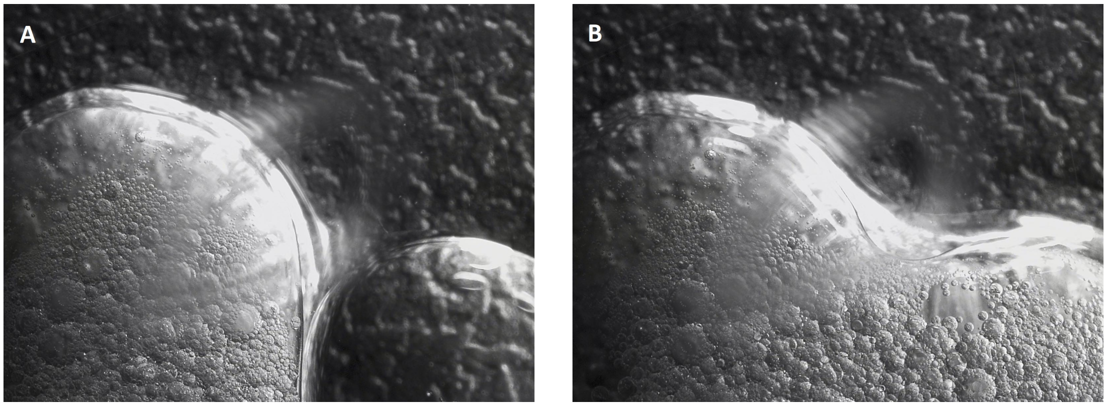

Figure S. 2: Consecutive microscope acquired frames of the coalescence test. (A) DPBS droplet (right) positioned close to the droplet of emulsion (left); (B) Occurred colescence between the two droplets. This allows to consider the emulsion as oil-in-water (O/W) type. To verify this result the same test has been performed positioning an oil droplet close to a droplet of emulsion. In this case, these two phases di not coalesce, confirming that the continuous phase of the emulsion is the DPBS solution.

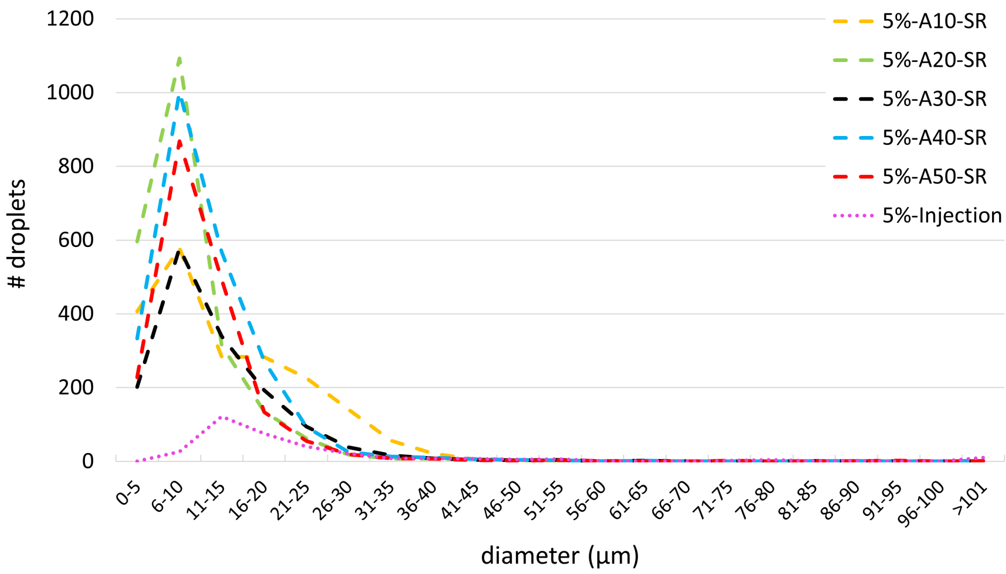

Figure S. 3: Comparison of droplets size distributions (in number of droplets) of emulsions obtained through SR experiments and with 5% of albumin concentration for all the investigated rotation amplitudes. Different color bars correspond to different imposed amplitudes. In the figure the droplets size distribution of drops observed after the injection phase is also reported.

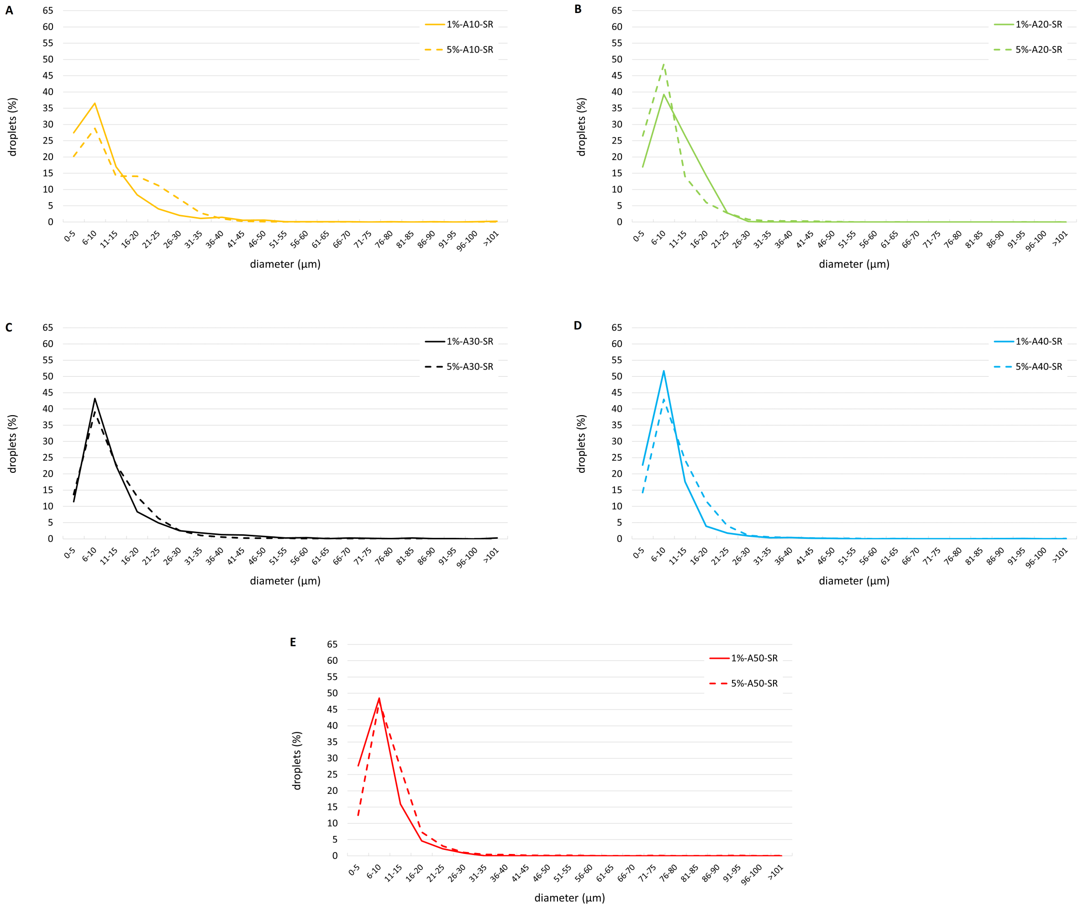

Figure S. 4: Comparison of droplets dimension distribution (in %) related to SR experiments with albumin concentration at 1% (continuous lines) and at 5% (dashed lines). Each panel reports the results obtained by imposing the same saccadic amplitude: (A) A=10°, (B) A=20°, (C) A=30°, (D) A=40°, (E) A=50°. Results obtained with the two albumin concentrations resulted very similar for all the imposed rotation amplitudes.

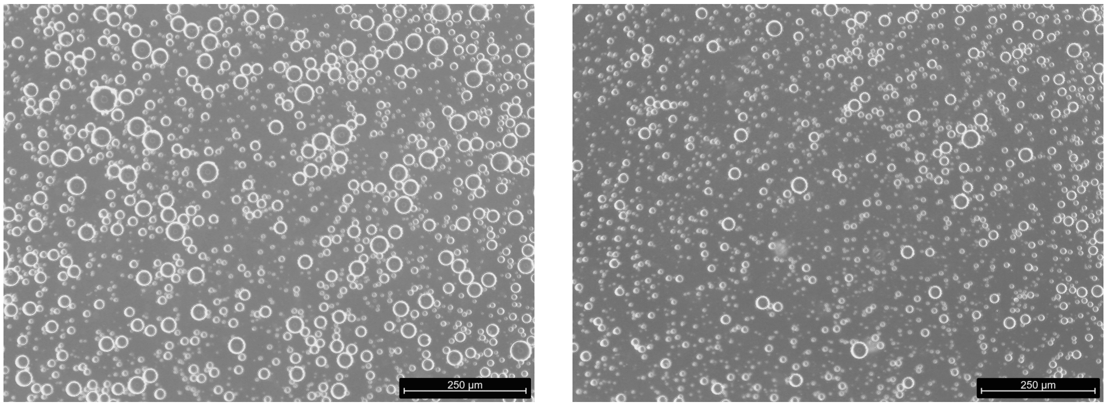

Figure S. 5: Example of microscope images of emulsions obtained through HR experiments with A=50°. A significantly larger area occupied by SO droplets for each image can be observed with respect the images acquired with lower HR amplitudes for the same albumin concentration.
